# In an older‐age vascular cohort, carotid stenosis is associated with processing speed and executive function cognitive deficits, which correlate with p‐tau217

**DOI:** 10.1002/alz.70893

**Published:** 2025-11-17

**Authors:** Summan Zahra, Scott R. French, Juan C. Arias, Madeline Ally, Haley Wiskoski, Cris Escareno, Emma Heitkamp, Franchell Vazquez, Madison Hillis, Gavin Culwell, Karthik Ainapurapu, Caronae M. Howell, Kevin Johnson, Deniz Karakay, Karthik Rayasam, Lindsay Taylor, Cody Kraemer, Luis Leon, Scott Berman, Federico Yanquez, Joshua Balderman, Joseph Sabat, Olivia Hung, Layla Lucas, Edward J. Bedrick, Francesca Vitali, Raza Mushtaq, Maria Altbach, Theodore P. Trouard, Ali Bilgin, Eric M. Reiman, Gene E. Alexander, Craig C. Weinkauf

**Affiliations:** ^1^ The Division of Vascular Surgery University of Arizona Tucson Arizona USA; ^2^ Department of Psychology University of Arizona Tucson Arizona USA; ^3^ Department of Biomedical Engineering University of Arizona Tucson Arizona USA; ^4^ Department of Medical Imaging University of Arizona Tucson Arizona USA; ^5^ Department of Electrical and Computer Engineering University of Arizona Tucson Arizona USA; ^6^ Department of Radiology Creighton University School of Medicine, Phoenix Health Sciences Phoenix Arizona USA; ^7^ Pima Heart and Vascular Tucson Arizona USA; ^8^ Department of Cardiovascular Medicine, Sarver Heart Center University of Arizona College of Medicine Tucson Arizona USA; ^9^ Department of Epidemiology and Biostatistics, Mel and Enid Zuckerman College of Public Health University of Arizona Health Sciences Tucson Arizona USA; ^10^ Department of Neurology University of Arizona Tucson Arizona USA; ^11^ Center for Innovation in Brain Science University of Arizona Tucson Arizona USA; ^12^ Department of Neuroradiology Barrow Neurological Institute Phoenix Arizona USA; ^13^ BIO5 Institute University of Arizona Tucson Arizona USA; ^14^ Evelyn F. McKnight Brain Institute University of Arizona Tucson Arizona USA; ^15^ Banner Alzheimer's Institute Phoenix Arizona USA; ^16^ Department of Psychiatry, Neuroscience and Physiological Sciences Graduate Interdisciplinary Programs University of Arizona Tucson Arizona USA; ^17^ Arizona Alzheimer's Consortium Phoenix Arizona USA

**Keywords:** ADRD biomarkers, Alzheimer's disease, asymptomatic carotid disease, cognition, cognitive domains, neurocognitive impairment, p‐tau217

## Abstract

**INTRODUCTION:**

Asymptomatic extracranial carotid atherosclerotic disease (aECAD) has been associated with Alzheimer's disease (AD), but the earliest cognitive deficits in this hallmark vascular population are largely unknown.

**METHODS:**

A total of 182 participants 50–85 years of age with ≥2 vascular comorbidities with/without aECAD were evaluated prospectively using a neurocognitive battery and compared to vascular comorbidities, percent stenosis, apolipoprotein E (APOE) *ε*4 status, and plasma phosphorylated tau (p‐tau217). Dementia, stroke < 6 months, and neurological disorders were exclusionary.

**RESULTS:**

aECAD was associated with significantly worse scores in processing speed (*β* = −0.19, *p *= 0.004) and executive function (*β* = −0.20, *p *= 0.009) domains after controlling for age, sex, education, race, and ethnicity. From these, a carotid cognitive index was created, which correlated with p‐tau217 (*β* = −0.29, *p *< 0.001), accounting for demographics, vascular comorbidities, white matter lesions, and *APOE* ε4 status.

**DISCUSSION:**

These data identify specific cognitive deficits associated with carotid stenosis and build further impetus to understand how vascular‐related cognitive deficits contrast and complement the classic memory deficits of AD.

## 1 INTRODUCTION

There is growing awareness that vascular comorbidities contribute to cognitive impairment and Alzheimer's disease (AD) risk.^1^ Vascular risk factors such as hypertension, hyperlipidemia, smoking, and diabetes are well known for their negative associations with cognitive function.^2^, ^3^, ^4^ In addition, vascular diseases such as carotid atherosclerosis, cerebral small vessel disease, stroke, and coronary artery disease also play a significant role in the pathogenesis of dementia.^1^, ^5^, ^6^, ^7^ Vascular contributions to cognitive impairment and dementia (VCID) are particularly important because they are often preventable/modifiable, which increases their relevance in AD‐related clinical management.^3^, ^8^ VCID‐related neurocognitive deficits encompass various domains, including memory with preponderance of attention, executive function, and processing speed domains.^8^, ^9^ However, VCID work has focused primarily on cerebral small vessel disease, vascular risk factors such as hypertension, and stroke‐related cognitive decline.^10^

Asymptomatic extracranial carotid atherosclerotic disease (aECAD) is a relevant cerebrovascular disease because it is associated with a 22% increase in AD risk^7^ and it occurs in 10% or more of the population 60 years of age or older.^11^ Studies evaluating the impact of aECAD on cognitive function have generally found significant associations with cognitive deficits, but have had significant limitations. These limitations include (1) a lack of internal control populations for those with aECAD, which is important because vascular risk factors present in patients with aECAD have been associated independently with neurocognitive changes; (2) reliance on the Mini‐Mental State Examination (MMSE) and Montreal Cognitive Assessment (MoCA)^12^, ^13^, ^14^, ^15^ without evaluation of the earliest cognitive domains to be affected; and (3) inconsistent accounting for age, sex, and education, which are highly important factors that affect neurocognitive testing. Identifying early cognitive differences in patients at risk for AD provides an opportunity to develop a window for therapeutic interventions.^16^, ^17^ Although tests such as the MMSE and MoCA have been validated as robust cognitive screening tools and utilized for a quick, standardized assessment in large epidemiological studies and clinical trials, neurocognitive test batteries, such as the National Alzheimer's Coordinating Center (NACC) Uniform Data Set (UDS), enable a more in‐depth understanding of cognitive performance across multiple cognitive domains^18^ and potential associations with neuropathological outcomes, particularly in AD.^19^, ^20^ From a clinical perspective, our recent work showed that treatment of aECAD mitigates AD risk.^21^ However, the current treatment guidelines for aECAD do not involve evaluation or treatment for cognitive outcomes.^22^, ^23^ Thus, identifying specific neurocognitive testing strategies for patients with aECAD at risk for AD is essential for future clinical trials and the development of early dementia risk mitigation strategies.

Markers of in vivo AD pathology present an additional opportunity to identify early disease changes. Blood‐based biomarkers provide a more scalable approach to detect such disease effects; among these, plasma phosphorylated tau‐217 (p‐tau217) has shown the highest diagnostic accuracy for detecting early AD pathology.^24^ Moreover, longitudinal changes in p‐tau217 levels are associated with decreased cognition in preclinical AD,^25^ highlighting that plasma p‐tau217 may provide important insights into the understanding of pathological AD progression. Plasma p‐tau217 has been found to be associated with cognitive dysfunction, with studies showing a stronger correlation with memory deficits.^26^ However, evaluation of p‐tau217 within vascular cohorts and its association with vascular‐related cognitive deficits is not well established.

Here, we first examined the neurocognitive status in a vascular risk cohort with and without aECAD using a standard neuropsychological battery to identify specific cognitive measures associated with aECAD. The neurocognitive measures with the strongest association with aECAD were then combined to create a “carotid cognitive index” for longitudinal assessment of cognition within this and related vascular cohorts. This cognitive index was then validated against plasma p‐tau217 to examine how these cognitive measures correlate with AD pathology in a vascular cohort. Improved understanding of vascular disease pathways involved in cognitive impairment and dementia may be a step toward addressing the medical and surgical needs of this large, at‐risk population.

## 2 METHODS

### 2.1 Study population

The first 182 participants enrolled in the prospective Carotid and Mind (CAM) clinical study were used for this analysis. CAM is a longitudinal observational study that evaluates the effect of vascular risk comorbidities on cognitive impairment and AD risk. Adults 50–85 years of age were recruited from vascular surgery/cardiology academic and community health clinics in Tucson, Arizona, from 2022 to 2024. Individuals were eligible if they had a clinical diagnosis of asymptomatic extracranial carotid atherosclerotic disease (aECAD) and/or at least two vascular risk factors, including smoking, hypertension, hyperlipidemia, or diabetes mellitus. aECAD was defined as having carotid stenosis >50% without a history of stroke or transient ischemic attack (TIA) in the last 6 months. In addition, brain magnetic resonance imaging (MRI) of all subjects was confirmed by a neuroradiologist (R.M.) for the absence of large cortical or subcortical infarcts. Exclusion criteria included a clinical diagnosis of dementia, major depression, neurological disorders, end‐stage renal disease, heart failure, or terminal cancer. Demographic information and past medical history were determined based on participants’ self‐reports (reviewed by study coordinators with participants) and medical records review. All study procedures were approved by the University of Arizona Institutional Review Board. All participants gave written informed consent.

### 2.2 MRI acquisition

A 3.0T Magnetom Siemens Skyra was utilized for both cerebral white matter lesion (WML) and carotid stenosis evaluation. A three‐dimensional (3D) T2‐weighted fluid‐attenuated inversion recovery (FLAIR) (1.0 × 1.0 × 1.2mm^3^) pulsed sequence was acquired with a 32‐element head radio‐frequency coil. Percent carotid stenosis was evaluated using a specialized carotid radio‐frequency coil.^27^ The evaluation was based on bright‐blood angiographic 3D time‐of‐flight (0.6 × 0.6 × 1mm^3^) and dark‐blood 2D T1‐weighted (in‐plane resolution = 0.6 × 0.6mm^2^; slice thickness = 2.0 mm) techniques (imaging parameters are provided in Table S1).

### 2.3 White matter lesion (WML) volume quantification and Fazekas grading

The WML volumes (mm^3^) were segmented using our in‐house developed software, a 3D orthogonal deep‐learning convolutional neural network.^28^ WML volumes were also correlated with visually graded WML, Fazekas (on a scale of 0–3), showing a strong correlation (*r* = 0.88, *p* < 0.001; Figure S1. WML volume data were missing for some participants (*n* = 18) who had contraindications to undergo MRI or who could not complete the scan session. Fazekas scoring was performed blindly by a trained neuroradiologist (R.M.) based on standard techniques.^29^, ^30^

### 2.4 Carotid stenosis evaluation

The final percent stenosis was adjudicated by a panel of three experts following North American Symptomatic Carotid Endarterectomy Trial (NASCET) criteria.^31^ Higher percent stenosis of the two carotid arteries was chosen for analysis. Percent carotid stenosis was used as a continuous measure of aECAD severity.

### 2.5 Neurocognitive testing

The neuropsychological battery was designed to evaluate older adults at risk for dementia, which included the NACC UDS, version 3.0.^32^ Adjunctive measures included the Stroop Color andWord Interference Test,^33^ the Rey Auditory Verbal Learning Test (RAVLT),^34^ and the Wechsler Adult Intelligence Scale, Fourth Edition (WAIS‐IV)^35^ Symbol Search and Coding Subtests. The cognitive tests were grouped into domains following prior work:^19^, ^33^, ^34^, ^35^

1. General Cognition: MoCA (Total Score)^36^
2. Memory: Immediate and Delayed Craft Story Recall (Paraphrase Scoring), Delayed Benson Figure Recall, and RAVLT Short and Long Delay Trials (A6 and A7; Total Correct)
3. Language: Category Fluency (Animals & Vegetables), Multilingual Naming Test (MINT) (Total Score), and Verbal Fluency (F & L Total Correct)
4. Executive Function: Trail Making Test Part B (Trails B), Backward Number Span (Total Correct), and Stroop Color andWord Interference (Total Correct)
5. Visuospatial: Benson Figure Copy
6. Processing Speed: WAIS‐IV Symbol Search (Total Score), WAIS‐IV Coding (Total Correct), and Trail Making Test Part A (Trails A)

*Z*‐scores were calculated for raw test scores (*Z*‐score = (Test score – Mean test score)/Standard deviation).^37^ *Z*‐scores were averaged to compute composite measures for the above domains. Z‐scores of tests showing significant association with carotid stenosis severity were averaged to form a composite called the carotid cognitive index.^38^ For all statistical analyses involving Trails A and B, *Z*‐scores were multiplied by −1; thus, lower scores indicated worse performance.

### 2.6 Blood‐based biomarkers and apolipoprotein E (*APOE*) genotyping

Blood was drawn using lithium heparin Vacutainer tubes and transported immediately to the University of Arizona Biorepository for processing and storage at −80°C. Peripheral blood mononuclear cells were isolated according to published protocols.^39^ Samples underwent one freeze–thaw cycle prior to analysis. Plasma p‐tau217 and apolipoprotein E (*APOE*) genotypes were quantified through collaboration with the Biomarker Assay Laboratory at the National Centralized Repository for Alzheimer's Disease (NCRAD). *APOE* gene variants (ε2, ε3, ε4) were defined within our cohort by analysis of single‐nucleotide polymorphisms (SNPs) at rs429358 and rs7412. Plasma p‐tau217 was measured using the Quanterix Simoa ALZpath pTau217 HD‐X Advantage Kit (Quanterix, Billerica, MA, USA Cat. 104371, Lot. 999024). Plasma p‐tau217 levels were used as a continuous measure and also binarized according to published cutoffs (0.63 pg/mL) of positron emission tomography (PET) positivity for subsequent analyses.^24^

### 2.7 Statistical analysis

The *t*‐test or chi‐square test was used to test the significance of the differences on continuous and categorical variables. Pearson correlation was used to test associations between continuous variables. Non‐parametric tests were used when normality criteria were not met. Multivariable linear regression was used to assess the effects of carotid disease, age, sex, education, race, ethnicity, and vascular risk comorbidities on relevant neurocognitive outcomes and controlled for multiple comparisons by performing false‐discovery rate (FDR) correction. The carotid cognitive index was further validated using confirmatory factor analysis (CFA). Levels of p‐tau217 were log‐transformed prior to linear regression analyses due to skewness, and post log‐transformation was normally distributed. WML volumes were also transformed to a logarithmic scale by using the equation log (WML volume+0.001), taking into consideration skewed distribution and participants having no WML. β Estimates for all regression models were standardized, and models were tested for multicollinearity using the variance inflation factor (VIF), with a VIF <1.2 indicating no multicollinearity. A *p*‐value < 0.05 was considered statistically significant. All analyses were conducted in R version 4.3.1. and GraphPad Prism 10.

## 3 RESULTS

### 3.1 Demographics

**FIGURE 1:**
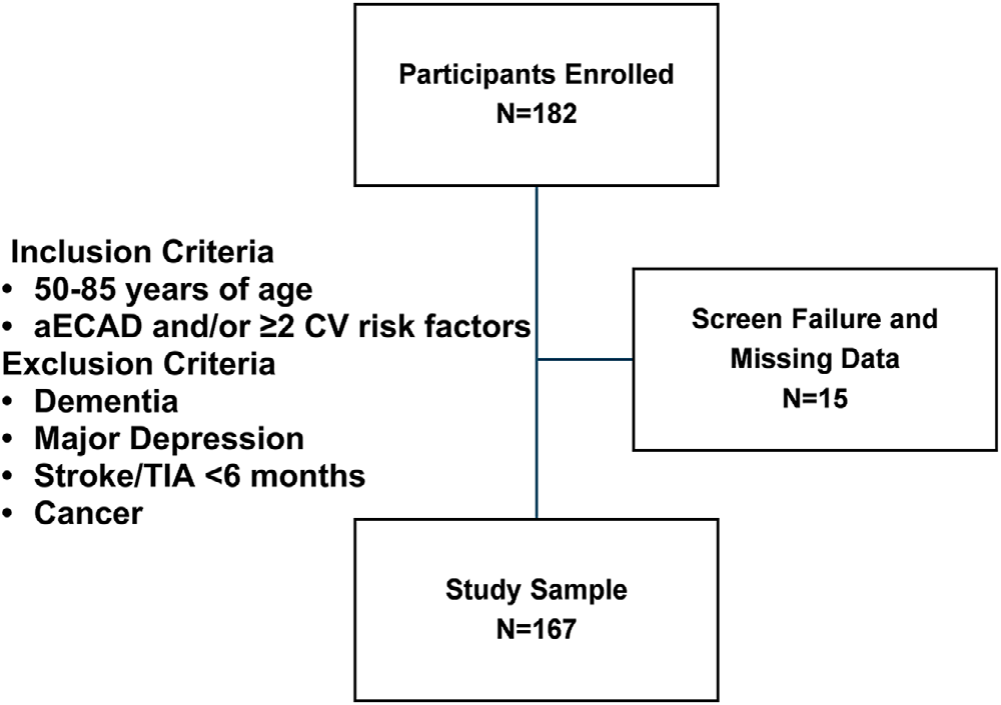
Flow diagram showing study sample. Inclusion and exclusion criteria are based on participants’ diseases and conditions diagnosed clinically/past medical history. aECAD, asymptomatic extracranial carotid atherosclerotic disease; CV, cardiovascular; TIA, transient ischemic attack.

Our study included 167 participants, excluding screening failures and missing data (Figure 1). Mean age was 72 ± 7 years, 57% were male, and education was 14.5 ± 2.9 years, with most participants having at least some college experience. Our sample was predominantly White (96%), with 11% of Hispanic/Latino ethnicity. Seventeen percent of the participants had a remote history of stroke, 37% had coronary artery disease, and 9% had a history of TIA. Prevalence of moderate (50%–69%) and severe (70%–99%) carotid stenosis was 22% and 29%, respectively. Thirteen percent had an occlusion in at least one carotid artery (Table 1).

**TABLE 1:** Demographic and clinical characteristics of study participants.

Characteristic	*N* = 167
**Age**	72 (7)
**Sex**	
Female	72 (43%)
Male	95 (57%)
**Race**	
White	159 (96%)
Asian	5 (3.0%)
Black	1 (0.6%)
Other	1 (0.6%)
Not reported	1 (0.6%)
**Ethnicity**	
Not Hispanic/Latino	144 (86%)
Hispanic/Latino	18 (11%)
Not reported	5 (3.0%)
**Education**	
≤12	49 (29%)
13–16	81 (49%)
≥17	37 (22%)
**Hypertension**	110(66%)
**Hyperlipidemia**	127(76%)
**Diabetes**	48 (29%)
**Smoking**	94 (56%)
**Coronary artery disease**	61 (37%)
**Chronic kidney disease**	13 (7.8%)
**Stroke**	28 (17%)
**Transient ischemic attack**	9 (5.4%)
**Carotid stenosis %**	
<50	60 (36%)
50–69	37 (22%)
70–99	49 (29%)
100	21 (13%)
***APOE ε4* allele**	43 (26%)
Missing	3

Continuous data are shown as mean (SD), whereas categorical data are shown as count (%).

Abbreviations: *APOE* ε4, apolipoprotein E ε4; SD, standard deviation.

### 3.2 Primary analysis

#### 3.2.1 Cognitive domains and aECAD

In unadjusted models, carotid stenosis showed a significant negative association with general cognition, memory, language, executive function, and processing speed, but not visuospatial domain. After controlling for age, sex, education, race, and ethnicity, increased severity of carotid stenosis was significantly associated with worse processing speed (*β *= −0.20, *p *= 0.004, *P_(FDR_*_) _= 0.02) and executive function (*β *= −0.19, *p *= 0.009, *p_(FDR_*_) _= 0.03) cognitive domain scores and not general cognition, memory, or language domains (Table 2).

**TABLE 2:** Linear regression analysis estimating the effect of percent carotid stenosis on neurocognitive domains and tests.

	Unadjusted			Adjusted^a^			
Outcomes	*𝛃*	95% CI	*p*	*𝛃*	95% CI	*p*	*p_(FDR)_*
Neurocognitive domains							
General cognition^b^	−0.22	−0.37 – −0.07	**0.004**	−0.08	−0.22 – 0.05	0.21	0.30
Memory	−0.19	−0.34 – −0.03	**0.02**	−0.08	−0.23 – 0.06	0.25	0.30
Language	−0.21	−0.36 – −0.06	**0.006**	−0.11	−0.26 – 0.04	0.14	0.28
Executive function	−0.29	−0.44 – −0.14	**<0.001**	−0.19	−0.33 – −0.05	**0.009**	**0.03**
Visuospatial	−0.07	−0.22 – 0.09	0.38	−0.08	−0.24 – 0.08	0.35	0.35
Processing speed	−0.32	−0.47 – −0.18	**<0.001**	−0.20	−0.34 – −0.07	**0.004**	**0.02**
**Neurocognitive tests within significant domains**							
Executive function							
Trails B^c^	−0.22	−0.37 – −0.07	**0.005**	−0.13	−0.28 – 0.01	0.07	0.11
Backward number span	−0.16	−0.31 – −0.01	**0.04**	−0.11	−0.27 – 0.05	0.16	0.16
Stroop C‐W interference	−0.32	−0.47 – −0.17	**<0.001**	−0.21	−0.35 – −0.07	**0.003**	**0.009**
Processing speed							
WAIS coding	−0.36	−0.50 – −0.22	**<0.001**	−0.26	−0.40 – −0.12	**<0.001**	**<0.001**
WAIS symbol search	−0.22	−0.37 – −0.07	**0.005**	−0.10	−0.25 – 0.04	0.15	0.16
Trails A^c^	−0.28	−0.42 – −0.13	**<0.001**	−0.21	−0.36 – −0.06	**0.007**	**0.01**

^a^ Adjusted for age, sex, education years, race, and ethnicity.

^b^ Montreal Cognitive Assessment (MoCA) score was used as a measure of general cognition. Memory score is composed of immediate craft story recall (paraphrase scoring), delayed craft story recall (paraphrase scoring), total score for delayed drawing of the Benson figure, Rey Auditory Verbal Learning Test (RAVLT) short and long delay trials (A6 and A7). Language score is composed of category fluency (animals), category fluency (vegetables), Multilingual Naming Test (MINT) total score, and number of correct F‐words and L‐words. Executive Function score is composed of Trail Making Test Part B, Backward Number Span Test (correct trials), Stroop Color andWord Interference Test. The visuospatial score is composed of the total score for the copy of the Benson figure visuospatial Index. The Processing Speed score is composed of the Wechsler Adult Intelligence Scale (WAIS) symbol search and coding, and the Trail Making Test Part A.

^c^ *Z*‐scores for Trail Making Parts A and B were multiplied by −1 so that lower scores indicated worse performance.

Abbreviations: β, standardized β‐coefficients, CI, confidence interval; *p*, *p*‐value; P_(FDR)_, false‐discovery rate corrected *p*‐value; Stroop C‐W interference, Stroop Color andWord Interference.

#### 3.2.2 Carotid cognitive index and aECAD

Association of neurocognitive tests within the processing speed and executive function domains with carotid stenosis, unadjusted and adjusting for age, sex, education, race, and ethnicity, was examined (Table 2). In adjusted models, performance on Stroop Color and Word Interference Test (*β *= −0.21, *p *= 0.003, *P_(FDR_*_) _= 0.009), WAIS‐IV Coding (*β *= −0.26, *p *< 0.001, *p_(FDR_*_) _< 0.001), and Trails A (*β *= −0.21, *p *= 0.007, *p_(FDR_*_) _= 0.01) decreased significantly with increased carotid stenosis percentage. Trails B (*β *= −0.13, *P *= 0.07) approached statistical significance, whereas WAIS‐IV Symbol Search (*β *= −0.10, *p *= 0.15) was not significantly associated (Table 2). Association of other individual neurocognitive tests in our battery with carotid stenosis was also assessed (Table S2).

**FIGURE 2:**
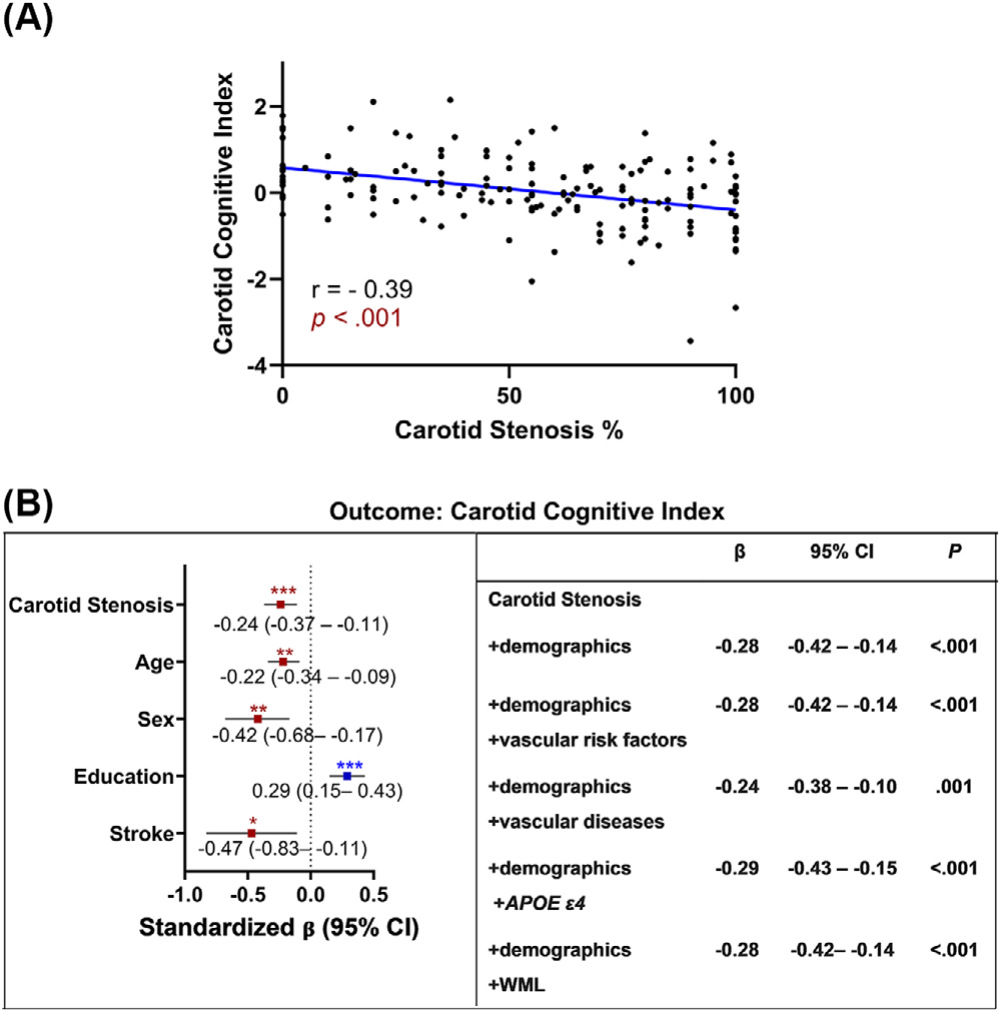
Carotid cognitive index is inversely associated with percent carotid stenosis. (A) Carotid cognitive index is a composite of WAIS‐Coding, Stroop Color andWord Interference, and Trail Making Test Part, combining domains of processing speed and executive function. The *r* value represents Pearson's correlation coefficient. (B) Multivariable linear regression analyses estimating the association of carotid stenosis with the carotid cognitive index. Model comprising all predictors with a significant association with carotid cognitive index (shown left) from the five models seen in the table (shown right). High carotid stenosis, older age, male sex, less education, and a history of stroke are all independently associated with significantly lower cognitive index scores. Demographics: age, sex, education, race, and ethnicity. Vascular risk factors: hypertension, hyperlipidemia, diabetes, and smoking. Vascular diseases: coronary artery disease, chronic kidney disease, transient ischemic attack, stroke. Sex: female [Reference]. *APOE* ε4, apolipoprotein E ε4 allele; *β*, standardized *β* coefficients; CI, confidence interval; WML, white matter lesion. *** *p* < 0.001 **p < 0.01 *p < 0.05.

A composite carotid cognitive index was formed comprising the three tests most strongly associated with carotid stenosis—Trails A, Stroop Color andWord Interference Test, and WAIS‐IV Coding tests. This cognitive index inversely correlated with percent carotid stenosis (*β* = −0.39, *p* < 0.001) (Figure 2A). Multivariable linear regression models adjusting for age, sex, education, race, ethnicity, vascular risk factors/diseases, *APOE ε4* allele status, and WML volumes were performed. Increased carotid stenosis severity was associated with a significant decrease in carotid cognitive index in all models (*β *= −0.24, *p *= 0.001). In addition, being male, less educated, and having a remote history of stroke were all significantly associated with cognitive deficits. Other vascular risk factors (hypertension, hyperlipidemia, diabetes, and smoking), diseases (coronary artery disease, chronic kidney disease, TIA), and *APOE ε4* allele status and WML volumes did not show a significant association (Figure 2B). Similar results were seen when Fazekas scores were used instead of WML volumes in the regression model (Table S3).

**FIGURE 3:**
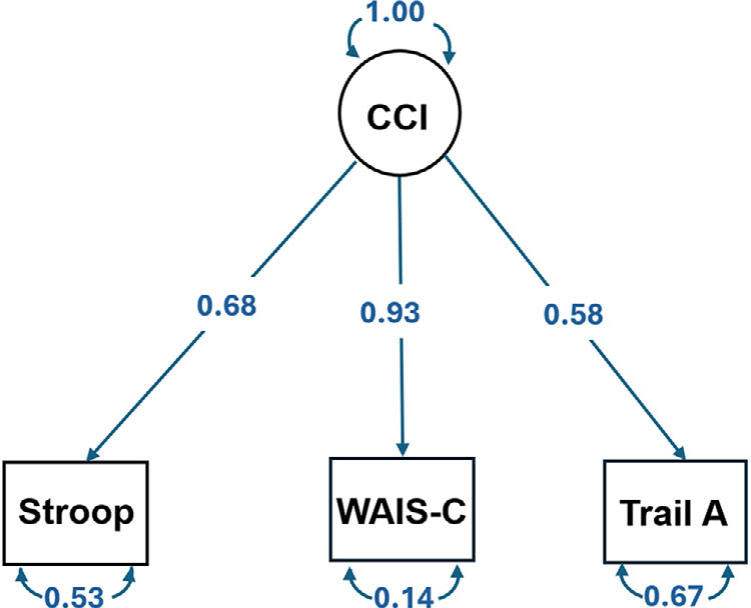
CFA diagram of latent variable representing carotid cognitive index (CCI). Note: RMSEA = 0.00; CFI = 1.00; TLI = 1.00; SRMR = 0.00. All indicators loaded significantly onto the factor at *p* < 0.001. Values are standardized estimates. For all three tests, Stroop Color andWord Interference (Stroop), WAIS‐IV Coding (WAIS‐C), and Trail Making Test Part A (Trails A), factor loadings represent standardized linear regression coefficients. Following conventional SEM notation, variables in squares were observed and measured, and variables in circles represent unmeasured latent variables. CFA, confirmatory factor analysis, RMSEA, root mean square error of approximation; CFI, Comparative Fit Index; SEM, Structural Equation Modelling; SRMR, standardized root mean square residual; TLI, Tucker Lewis Index.

In addition to the mean composite, a single‐factor confirmatory factor analysis (CFA) model for the carotid cognitive index showed that all three tests, WAIS‐IV Coding, Trails A, and Stroop Color andWord Interference Test, loaded significantly (0.93, 0.58, 0.68, respectively, *p *< 0.001). This suggests that these measures of processing speed and executive function contribute significantly to the carotid cognitive index, with WAIS‐IV Coding showing the strongest association (Figure 3).

Patients in this study were required to be asymptomatic for stroke or TIA within the past 6 months, but 28 (17%) reported a remote history of stroke. In addition to controlling for stroke in regression models, a sensitivity analysis was performed excluding these participants. The effect of carotid stenosis on carotid cognitive index remained significant in both unadjusted (*β *= −0.36, *p *< 0.001) and adjusted linear regression models (*β *= −0.23–−0.30, *p *< 0.01) after exclusion of stroke (Table S4).

### 3.3 Secondary analysis

#### 3.3.1 Carotid cognitive index validation with p‐tau217

**FIGURE 4:**
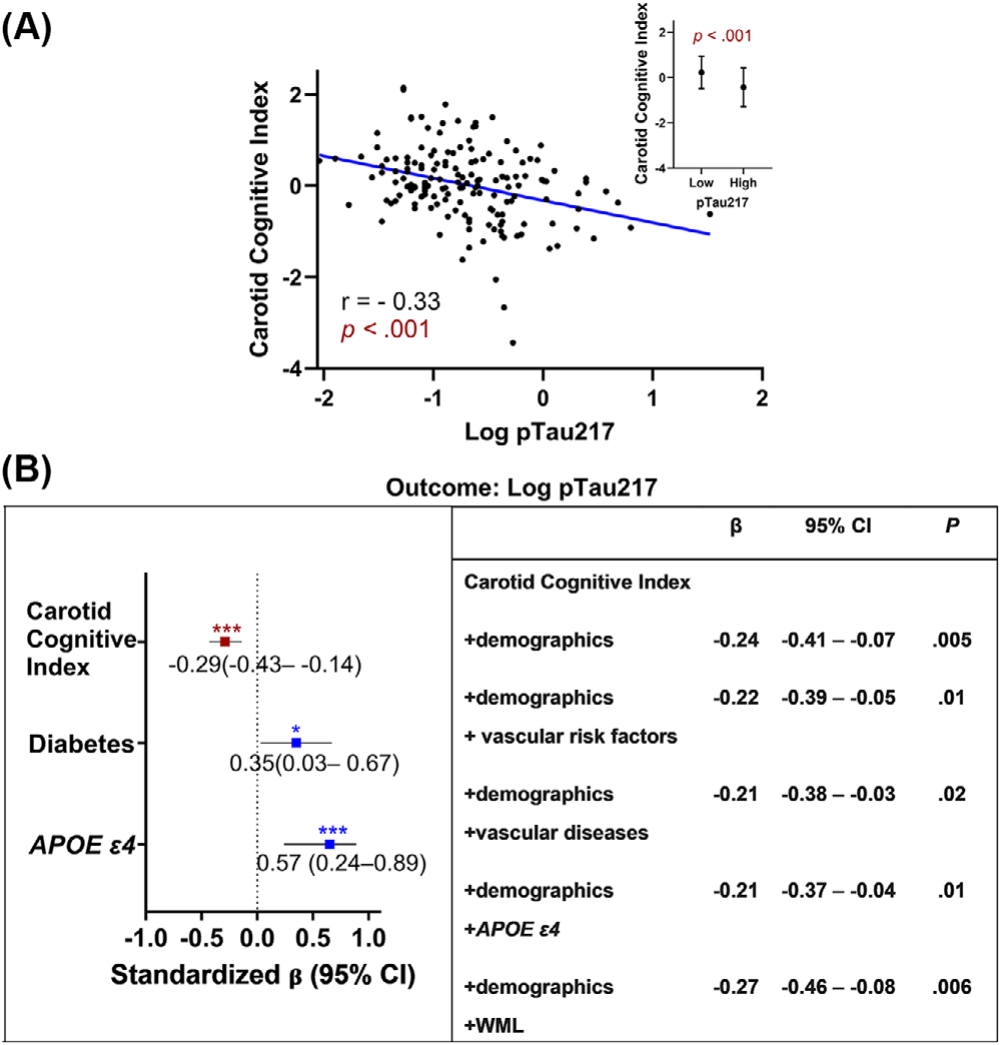
Carotid cognitive index is significantly associated with AD biomarker, plasma p‐tau217. (A)The *r* value represents Pearson's correlation coefficient. Inset: Mean carotid cognitive index is significantly lower in the high p‐tau217 (>0.63 pg/mL) as compared to the low p‐tau217 (≤0.63 pg/mL) group; cutoff is based on established values.^24^ Mean differences were evaluated using the Wilcoxon test. (B) Multivariable linear regression analyses estimating the association of carotid cognitive index with p‐tau217 levels, adjusting for age, sex, education, race, ethnicity, vascular risk factors/diseases, *APOE ε4* allele, and WML volumes. ​Model comprising all significant predictors (shown left) associated with p‐tau217 levels. A worse carotid cognitive index score, the presence of diabetes, and the presence of the *APOE ε4* allele are associated with increasing p‐tau217 level. This association remained significant after inclusion of potential confounders (shown right). Demographics: age, sex, education, race, and ethnicity. Vascular risk factors: hypertension, hyperlipidemia, diabetes, and smoking. Vascular diseases: coronary artery disease, chronic kidney disease, transient ischemic attack, and stroke. *APOE* ε4, apolipoprotein E ε4 allele; *β*, standardized *β* coefficients; CI, confidence interval; WML, white matter lesion. *** *p* < 0.001 **p < 0.01 *p < 0.05.

The carotid cognitive index negatively correlated with p‐tau217 levels (*r *= −0.33, *p *< 0.001), and mean carotid cognitive index scores were significantly lower in the high p‐tau217 (>0.63 pg/mL) group compared to the low p‐tau217 (≤0.63 pg/mL) group (*p *< 0.001; Figure 4A). Multivariable linear regression models showed that this index was significantly associated with decreased p‐tau217 levels independent of age, sex, education, race, or ethnicity (*β *= −0.24, *p *= 0.005), *APOE* ε4 status (*β *= −0.21, *p *= 0.01), vascular risk factors (*β *= −0.22, *p *= 0.01), vascular diseases (*β *= −0.21, *p *= 0.02), or WML volumes (*β *= −0.27, *p *= 0.006), (Figure 4B). Association of cognitive and p‐tau217 was similar when Fazekas was used instead (*β *= −0.27, *p *= 0.006), (Table S5). The presence of the *APOE ε4* allele was associated with high p‐tau217 levels (*β *= 0.57, *p *< 0.001) (Figure 4B). When cognitive domains were assessed separately, domains of executive function and processing speed showed a significant association with p‐tau217 levels after adjusting for age, sex, education, race, and ethnicity (Table S6).

## 4 DISCUSSION

In this study, a cohort of 167 participants with vascular comorbidities with or without aECAD were prospectively enrolled to study the association of aECAD with cognition. These data demonstrated that aECAD was associated with significantly poorer scores for neurocognitive domains of processing speed and executive function after adjusting for age, sex, education, race, ethnicity, vascular comorbidities, and WML volumes. The individual neurocognitive tests that demonstrated a statistically significant association with aECAD severity after adjustment for multiple comparisons were used to create a “carotid cognitive index,” which was found to be significantly associated with the AD biomarker, p‐tau217.

In the context of previously published and ongoing related studies, this work involved non‐demented community‐dwelling participants with vascular comorbidities. The cohort had a mean age of 72 years and was 43% female. Vascular risk factors including hypertension (66%), diabetes (29%), hyperlipidemia (76%), and smoking (56%) were within expectations for a vascular cohort, such as the Framingham Heart Study.^40^ This cohort is, therefore, likely representative of vascular populations in the United States except for higher rates of carotid atherosclerosis (as expected) and slightly higher Hispanic (11%) and lower Asian (3%) and Black (0.6%) participants.

Prior work demonstrates no clear agreement on whether aECAD is associated with specific neurocognitive deficits and which cognitive domains are affected earliest.^41^, ^42^, ^43^, ^44^ Two key studies evaluating neurocognitive deficits in patients with aECAD found that memory is primarily affected.^42^, ^43^ The largest of these involved a sub‐population of the Carotid Revascularization and Medical Management for Asymptomatic Carotid Stenosis Trial (CREST‐2), which used baseline visits in 786 stroke‐free participants with aECAD (>70% carotid stenosis); their neurocognitive status was compared to the normalized Reasons for Geographic and Racial Differences in Stroke (REGARDS) population.^42^ The CREST‐2 data have some limitations making its interpretation challenging and less comparable to our work: (1) a limited telephone‐administered neurocognitive battery was used not including tests assessing processing speed; (2) sex was not controlled for in the analyses performed, which is a highly relevant because sex affects performance on cognitive tests^45^ and is skewed in aECAD patients; and (3) there was no internal control cohort within the CREST‐2 study (i.e., participants without carotid stenosis). Despite these considerations, this and several other studies have consistently demonstrated that aECAD has an independent association with cognitive impairment,^13^, ^14^, ^15^, ^42^, ^43^, ^46^ although the specificity of the earliest affected cognitive domains remains unanswered. The current work using an in‐person, 90‐min neurocognitive battery provided a broad evaluation of cognition, including general cognition, memory, language, visuospatial function, processing speed, and executive function. This study has the benefit of internal controls (those without carotid disease) and addresses key potential confounders, including age, sex, education, race, ethnicity, other vascular comorbidities, and WML volumes. This study also evaluated AD biomarkers and *APOE ε4* allele status to complement neurocognitive assessment, providing a key and novel understanding of how observed neurocognitive performance was associated (or not) with AD pathology in a vascular‐rich carotid disease cohort.

Within the context of AD pathology, it is notable that episodic memory deficits are widely considered the hallmark of preclinical AD.^47^, ^48^ We found that memory (with testing focused on episodic memory) was not significantly associated with aECAD severity. This may suggest that the cognitive impairment seen in this cohort and represented in the carotid cognitive index is distinct compared to the more classic cognitive decline associated with early AD. Previous research has suggested that domains of executive function and attention may be predictive of AD development later in life; however, the majority of the studies focus only on global and episodic memory assessment.^49^, ^50^, ^51^, ^52^, ^53^ Our data demonstrated that WAIS‐IV Coding, a measure of processing speed, showed the highest correlation with carotid stenosis and the strongest contribution to the carotid cognitive index among the three tests. Decreased processing speed has been associated with aging^54^ and multiple dementia subtypes, including vascular dementia.^55^, ^56^, ^57^ Within our vascular cohort, the association of processing speed with carotid disease severity was independent of age, vascular comorbidities, and WML volumes. It has been suggested that performance on tasks in various cognitive domains relies on information processing,^58^ and its early decline may lead to differences in other domains. Thus, it may be that integration of processing speed with deficits spanning other cognitive domains, such as memory, will be seen over time in this cohort. Further testing of these cognitive features in patients with and without vascular comorbidities will benefit our understanding of how vascular diseases may contribute to cognitive changes across dementia types.

To further understand the relevance of carotid disease and observed cognitive deficits in the context of Alzheimer's pathology, we evaluated plasma p‐tau217. We found that worse scores on our carotid cognitive index were significantly associated with higher p‐tau217 levels (*β* = −0.33, *p* < 0.001). This association remained significant after adjusting for age, sex, education, race, ethnicity, *APOE* ε4 allele status, stroke, other vascular risk comorbidities, and WML volumes. Plasma p‐tau217 is a strong predictor of cognitive decline in AD,^59^ and our recent work showed that within a vascular cohort, 55% had elevated plasma p‐tau217 levels, which is often underdiagnosed in cardiovascular populations.^60^ Association of carotid cognitive index with p‐tau217 highlights and builds impetus to better understand how aECAD contributes to AD pathogenesis.

Stroke is associated with cognitive impairment and dementia.^5^, ^61^ Although only patients with “asymptomatic” carotid disease were recruited for this study, meaning participants did not have a stroke within 6 months, 17% still had a remote history of stroke. Regression analyses and sensitivity analyses excluding stroke patients (Table S3) confirmed that both stroke and aECAD have independent associations with the cognitive impairment seen in this cohort. Beyond stroke, other vascular comorbidities are associated with cognitive impairment.^2^, ^3^ In addition, WMLs, a marker of cerebral small vessel disease, have also been shown to be associated with neurocognitive deficits,^62^ but we found it surprising that no other vascular comorbidities or white matter disease significantly affected the cognitive outcomes in our regression models. Collinearity could explain this, but aECAD was not collinear with other evaluated vascular comorbidities (VIF <1.2). It may also be that many or most studies looking at VCID do not evaluate/control for carotid stenosis.^2^, ^3^, ^63^ As such, these data may be best interpreted that among patients with vascular comorbidities, aECAD (and stroke) has the earliest and/or strongest association with cognitive impairment.

This study has some limitations: (1) The carotid cognitive index results are limited to our carotid cohort, encouraging future studies to validate this tool in wider populations. (2) Plasma p‐tau217 has not been studied specifically in vascular populations, which makes the data novel but can limit our interpretation of published cutoff values for positivity. This limitation is mitigated by our use of p‐tau217 as a continuous variable, and also that it correlates with cognition. (3) Although we do control for race and ethnicity, this cohort is predominantly White, with 11% Hispanic and 86% non‐Hispanic, so interpreting these data with caution with regard to race and ethnicity is warranted.

In summary, this study identified processing speed and executive function as significantly affected cognitive domains in patients with aECAD, a hallmark vascular disease that increases AD risk. It also created a carotid disease cognitive index that correlated significantly with AD pathology and may be useful for future studies in this and related cohorts. Although patients with carotid disease are not regularly evaluated clinically for cognitive impairment or dementia, this work builds momentum for clinical management changes and future studies with a long‐term goal of dementia prevention.

## CONFLICT OF INTEREST STATEMENT

E.M.R. is a co‐founder advisor and shareholder of ALZpath (pTau217 capture antibody and assay), and a compensated scientific advisor to Alzheon, Denali, Cognition Therapeutics, Enigma, Retromer Therapeutics, and Vaxxinity. Although the Quanterix ALZpath assay was used to characterize p‐tau‐217 levels, he was not involved in the analysis of data. None of the other authors declare that they have conflicts of interest. Any author disclosures are in the Supporting Information.

## ETHICS APPROVAL

All experiments were conducted in accordance with the Declaration of Helsinki. All procedures are approved by the institutional review board at the University of Arizona (1606653257), and all participants, or their legal representatives provided written informed consent.

## CONSENT TO PARTICIPATE

All subjects or their legal representatives signed written informed consent.

## CODE AVAILABILITY

All analytic software is publicly available as described in the Methods section of this article.

## Supporting information



Supporting Information

Supporting Information

## Data Availability

The de‐identified data utilized in the analyses for this study will be made available upon reasonable request to the corresponding author (C.W.) and receipt of a signed data access agreement form.
