# Supplementary material for: In an older‐age vascular cohort, carotid stenosis is associated with processing speed and executive function cognitive deficits, which correlate with p‐tau217
Source: Alzheimers Dement. 2025 Nov 17;21(11):e70893. doi: 10.1002/alz.70893 (PMC12623130; doi:10.1002/alz.70893)
Supplement: Supplementary file 1 — Supporting Information [file ALZ-21-e70893-s002.docx]

**Supplementary Table 1: Imaging parameters for the two pulse sequences used in the evaluation of white mater lesions and carotid stenosis.**

| **Sequence** | **3D Time-of-Flight** | **2D T1-weighted Turbo Spin Echo** | **T2-weighted Fluid-Attenuated Inversion Recovery** |
| --- | --- | --- | --- |
| TR/TE (ms) | 21.0/3.7 | 710.0/9.1 | 4,800.0/441.0* |
| Resolution (mm^3^) | 0.63x0.63x1.0 | 0.63x0.63x2.0 | 1.0x1.0x1.2 |
| Number of Slices | 40 per/slab (4 slabs) | 30 | 160 |
| Flip angle (degrees) | 15 | 90/150 | VFL (SPACE) |
| Echo train length | - | 4 | 243 |
| Parallel imaging (GRAPPA) factor | 2 | 2 | 3 |
| Saturation bands | Superior | Inferior | None |

*Apparent TE119.0 ms. VFF, variable flip angle; SPACE, Sampling Perfection with Application-Optimized Contrasts Using Different Flip Angle Evolutions; TR, repetition time; TE, echo time; MS, millisecond

**Supplementary Figure 1. White matter lesion volumes correlate strongly with visually graded Fazekas.**


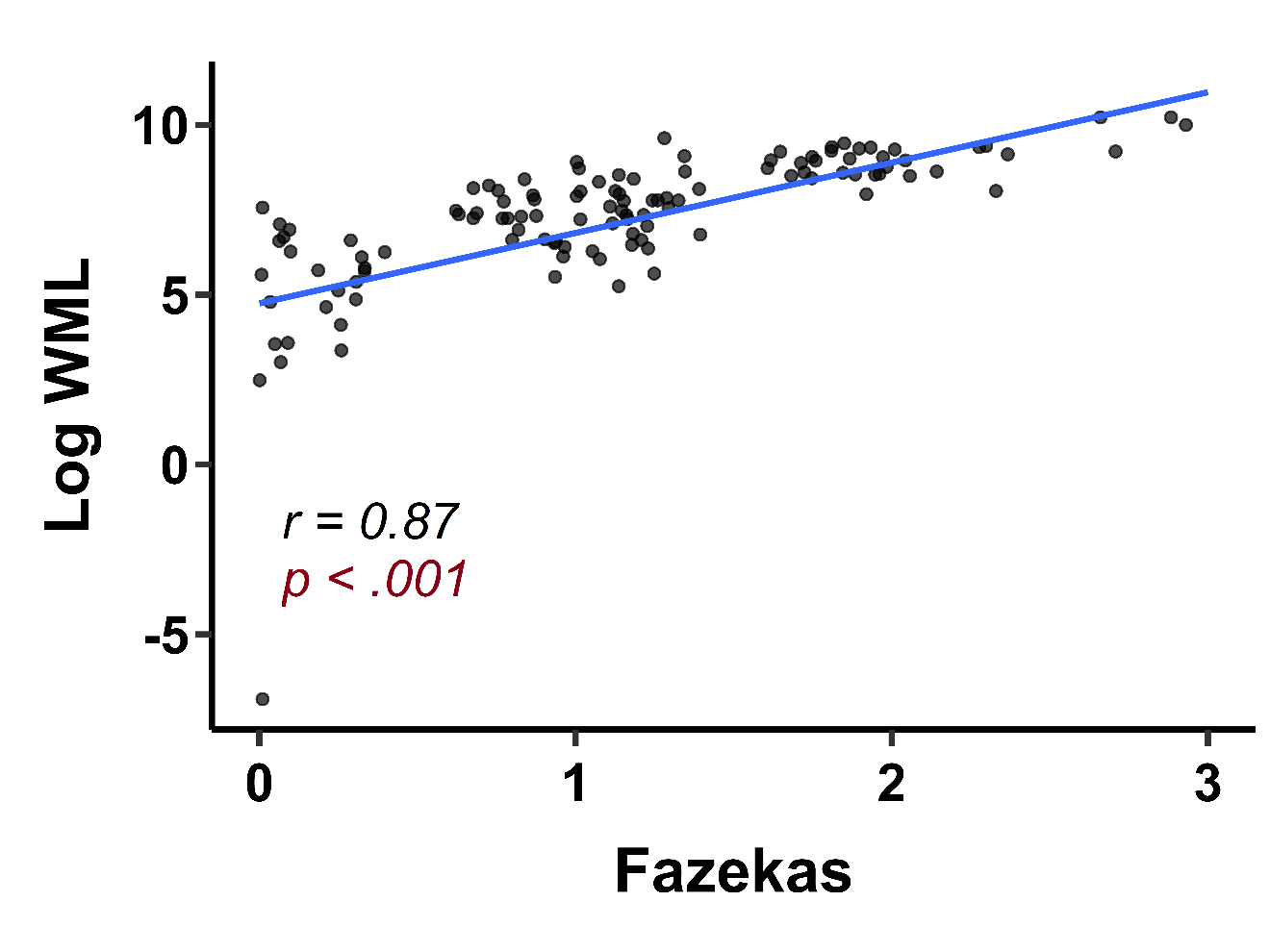


Abbreviations, WML, White matter lesion; r, correlation coefficient.

**Supplementary Table 2. Adjusted^a^ linear regression analysis estimating the effect of percent carotid stenosis on neurocognitive tests.**

| **Outcomes** | 𝛃 | **95% CI** | ***P*** | ***P_(FDR)_*** |
| --- | --- | --- | --- | --- |
| Craft Story Immediate | -0.11 | -0.26 – 0.05 | .17 | .34 |
| Craft Story Delayed | -0.07 | -0.22 – 0.09 | .39 | .50 |
| Benson Immediate | -0.07 | -0.23 – 0.09 | .36 | .50 |
| Benson Delayed | -0.10 | -0.26 – 0.06 | .22 | .37 |
| Category Fluency (Animals) | -0.07 | -0.23 – 0.08 | .34 | .50 |
| Category Fluency (Vegetables) | -0.12 | -0.28 – 0.03 | .12 | .34 |
| MINT | -0.03 | -0.19 – 0.12 | .68 | .72 |
| Verbal Fluency (F) | -0.10 | -0.25 – 0.04 | .17 | .34 |
| Verbal Fluency (L) | -0.09 | -0.24 – 0.06 | .23 | .37 |
| Forward Number Span | -0.06 | -0.22 – 0.10 | .44 | .53 |
| RAVLT Short Delay Trial | -0.03 | -0.19 – 0.12 | .67 | .72 |
| RAVLT Long Delay Trial | -0.02 | -0.18 – 0.14 | .83 | .83 |
| ^a^Adjusted for age, sex, education years, race, and ethnicity. Abbreviations: β, standardized β-coefficients; CI, confidence interval, P_(FDR)_, False discovery rate corrected P value; *P, P* value; MINT, Multilingual Naming Test; RAVLT, Rey Auditory Verbal Learning Test; Stroop C-W Interference, Stroop Color-Word Interference Test. | | | | |

**Supplementary Table 3. Linear regression model estimating the effect of carotid stenosis on carotid cognitive index adjusting for demographics and Fazekas.**

**Outcome: Carotid Cognitive Index**

|  | **β** | **95% CI** | ***P*** |
| --- | --- | --- | --- |
| Carotid Stenosis | -0.28 | -0.42- -0.14 | <.001 |
| Age | -0.28 | -0.42- -0.14 | <.001 |
| Sex [Male] | -0.38 | -0.65- -0.12 | .005 |
| Education | 0.23 | 0.09- 0.37 | .002 |
| Race [White] | 0.31 | -0.38- -0.99 | .38 |
| Ethnicity [Hispanic/Latino] | -0.03 | -0.47- 0.40 | .89 |
| Fazekas | 0.02 | -0.12- 0.16 | .78 |
| Abbreviations: β, standardized β coefficients; CI, confidence interval; *P, P* value | | | |

**Supplementary Table 4.** **Sensitivity analysis after exclusion of participants (N=28) with history of stroke (greater than six months)**

**Outcome: Carotid Cognitive Index**

|  | **β** | **95% CI** | ***P*** |
| --- | --- | --- | --- |
| Carotid Stenosis | -0.36 | -0.52- -0.20 | <.001 |
| +demographics | -0.24 | -0.39- -0.09 | .002 |
| *+*demographics+ vascular risk factors | -0.23 | -0.39- -0.08 | .004 |
| +demographics+ vascular diseases | -0.23 | -0.39- -0.08 | .004 |
| +demographics+ *APOE ε4* | -0.25 | -0.40- -0.10 | .001 |
| Demographics: age, sex, education, race, ethnicity. Vascular risk factors: hypertension, hyperlipidemia, diabetes, smoking. Vascular Diseases: coronary artery disease, chronic kidney disease, transient ischemic attack, stroke. Abbreviations: *APOE ε4*, Apolipoprotein ε4; β, standardized β coefficients; CI, confidence interval; *P, P* value | | | |

**Supplementary Table 5. Linear regression model estimating the association of carotid cognitive index and pTau217 adjusting for demographics and Fazekas.**

**Outcome: Log pTau217**

|  | **β** | **95% CI** | ***P*** |
| --- | --- | --- | --- |
| Carotid Cognitive Index | -0.27 | -0.45- -0.08 | .006 |
| Age | 0.19 | 0.02 - 0.37 | .03 |
| Sex | 0.19 | -0.14 - 0.52 | .26 |
| Education | -0.04 | -0.22 - 0.14 | .64 |
| Race | 0.50 | -0.37 - 1.38 | .26 |
| Ethnicity | -0.13 | -0.65 – 0.39 | .62 |
| Fazekas | -0.04 | -0.20 – 0.13 | .65 |
| Abbreviations: β, standardized β coefficients; CI, confidence interval; *P, P* value | | | |

**Supplementary Table 6: Linear regression analysis estimating the effect of pTau217 levels on neurocognitive domains.**

**Outcome: Log ptau217**

|  | **Adjusted^a^** | | | |
| --- | --- | --- | --- | --- |
| **Cognitive Domains** | **𝛃** | **95% CI** | ***P*** | ***P_(FDR)_*** |
| General Cognition^b^ | -0.25 | -0.42 – -0.07 | .006 | .02 |
| Memory | -0.01 | -0.18 – 0.17 | .92 | .92 |
| Language | -0.18 | -0.36 – -0.01 | **.04** | .06 |
| Executive Function | -0.20 | -0.37 – -0.03 | **.02** | **.04** |
| Visuospatial | -0.06 | -0.22 – 0.10 | .45 | .54 |
| Processing Speed | -0.25 | -0.42 – -0.08 | **.004** | **.02** |
| ^a^Adjusted for age, sex, education years, race, and ethnicity. ^b^MoCA score was used as a measure of general cognition. Memory score is composed of immediate craft story recall (paraphrase scoring), delayed craft story recall (paraphrase scoring), total score for delayed drawing of Benson figure, RAVLT short and long delay trials (A6 and A7). Language score is composed of category fluency (animals), category fluency (vegetables), MINT total score, number of correct F-words and L-words. Executive Function score is composed of trail making test part B, backward number span test (correct trials), Stroop Color-Word Interference Test. Visuospatial score is composed of total score for copy of Benson figure visuospatial Index. Processing Speed score is composed of WAIS symbol search and coding and trail making test part A. **^c^**Z- scores for Trail Making Part A and B were multiplied by − 1 so that lower scores indicated worse performance. **Abbreviations:** β, standardized β-coefficients**; CI, confidence interval; *P*, *P* value;** P_(FDR)_, false discovery rate corrected *P* value; Stroop C-W Interference, Stroop Color-Word Interference. | | | | |
